# Protocol for the SEHNeCa randomised clinical trial assesing Supervised Exercise for Head and Neck Cancer patients

**DOI:** 10.1186/s12885-023-10718-4

**Published:** 2023-03-24

**Authors:** M. Rodriguez-Arietaleanizbeaskoa, E Mojas Ereño, MS Arietaleanizbeaskoa, G. Grandes, A Rodríguez Sánchez, V. Urquijo, I Hernando Alday, M. Dublang, G. Angulo-Garay, J. Cacicedo, on behalf of the SEHNeCa group, Mario Rodriguez-Arietaleanizbeaskoa, Egoitz Mojas Ereño, Maria S. Arietaleanizbeaskoa, Gonzalo Grandes, Arturo Garcia-Alvarez, Aitor Coca, Nere Mendizabal, Olga del Hoyo, Javier García-Escobedo, Ángel Rodríguez Sánchez, Lucía Flores Barrenechea, Rebeca Sánchez, Virginia Urquijo, Luis Barbier Herrero, Goiztidi Díaz-Basterra, Javier Gómez-Suarez, Laura A Calles Romero, Natalia C. Iglesias-Hernandez, Iñigo Hernando Alday, Maddalen Dublang, Lina M. Ramirez-Garcia, Garazi Angulo-Garay, Silvia Dominguez-Martinez, Erreka Gil-Rey, Aitor Martinez-Aguirre, Borja Gutierrez-Santamaria, Jon Cacicedo

**Affiliations:** 1 Comprehensive Care Group for Patients With Chronic Diseases, Biocruces Bizkaia Health Research Institute, Plaza de Cruces 12, 48903 Barakaldo, Bizkaia Spain; 2 AFD Salud. Department of Physical Activity and Sport Sciences, Faculty of Education and Sport, University of Deusto, Bizkaia, 48007 Spain; 3 Primary Care Research Unit – Bizkaia, Basque Health Service (Osakidetza), General Directorate, Vitoria-Gasteiz, Spain; 4 Basurto Radiation Oncology Department, Avenida Montevideo 18, 48013 Bilbao, Bizkaia Spain; 5 Cruces Endocrinology Department, Plaza de Cruces S/N, 48903 Barakaldo, Bizkaia Spain; 6 Basurto Endocrinology Department, Avenida Montevideo 18, 48013 Bilbao, Bizkaia Spain; 7 Galdakao Endocrinology Department, Barrio Labeaga, 48960 Galdakao, Bizkaia Spain; 8 Department of Radiation Oncology, Group for Radiology and Physical Medicine in Oncology, Cruces University Hospital/Biocruces Bizkaia Health Research Institute, Barakaldo, 48903 Spain

**Keywords:** Supervised physical exercise, Head and neck cancer, Lean body mass, Quality of life, Radiotherapy, Sarcopenia

## Abstract

**Objectives:**

To evaluate the effectiveness of an innovative supervised exercise programme to mitigate the loss of lean body mass, functional capacity and quality of life in people with head and neck cancer, as well as to identify the optimal moment to apply it, before or after radiotherapy treatment, compared with the prescription of a physical activity plan carried out autonomously.

**Methods:**

Patients with squamous cell carcinoma of the head and neck (*n* = 144), treated with radiotherapy, will be randomly assigned to one of 3 comparison groups: pre-radiotherapy supervised exercise, post-radiotherapy supervised exercise and autonomous exercise, stratifying by human papillomavirus infection and previous surgery.

The exercise programme will be carried out in 36 sessions over 12 weeks, combining moderate and high intensity strength and aerobic exercises. The main outcome variable is the change in lean body mass at 6 months measured by bioimpedance, while secondary variables are functional capacity, symptoms, quality of life and adverse effects. Longitudinal generalised mixed models will be used for the analyses of the repeated measurements at 3, 6, and 12 months after baseline.

**Conclusions:**

The pilot study supports the feasibility and safety of the project. However, as the programme progressed, attendance at the sessions decreased. Strategies will be necessary for increasing attendance, as well as involving the patient in their recovery and other incentives. Follow-up after treatment to assess acute/late toxicity will enable us to know the response to both the exercise programme and its adherence.

**Trial registration:**

NCT04658706 Date and version identifier: March 1, 2023. Version 1.1

## Introduction

Head and neck cancer (HNC) was the seventh most frequently diagnosed cancer worldwide in 2020 [1]. The main risk factors that cause this type of cancer to develop are smoking, alcoholism, diet, sedentary lifestyle, obesity and human papillomavirus (HPV) infection, among others [2]. This unhealthy background added to both the disease and its treatments effects tremendously reduce cardiorespiratory capacity, lean body mass (through accelerated processes of cachexia and sarcopenia) bone mass, and provoke psychosocial distress, depression, pain, fatigue, and impaired cognitive function [3].

Between 30 and 50% of HNC patients have malnutrition and it has been estimated that they may experience 5–10% weight loss during radiotherapy treatment [4, 5]. Weight loss of more than 5% has been associated with a decrease in overall and specific survival for head and neck cancer. In addition, it is estimated that more than 70% of weight loss can be attributed to loss of lean body mass, which leads to a decrease in strength, functional capacity and quality of life [6]. Because of that lean body mass is considered an appropriate and feasible end point in clinical trials of HNC. Additionally, sarcopenia before and after treatment is an independent factor of poor prognosis associated with decreased survival in patients undergoing radiotherapy with curative intent [7]. Moreover, sarcopenia has recently been associated with increased acute toxicity (grade ≥ 3 dysphagia) [8] and decreased progression-free survival [9]. For this reason, it is deemed appropriate to perform body composition measurements as part of the diagnosis and evaluation of interventions for HNC [10–12].

Nutritional approach is a fundamental part of support for these patients. Nutritional interventions through oral supplements and dietary advice have shown short-term benefit in patients with HNC [13]. Patients with head and neck tumours represent a group with a high prevalence of malnutrition and with very specific side effects compared to other types of tumours, which can cause loss of lean body mass [8]. Most studies have explored the impact of either nutritional supplements or physical exercise (PE) separately. Therefore, it seems necessary to explore the benefit of nutritional intervention and PE jointly in these patients and their impact on lean body mass. Up to now, randomised studies that have assessed the impact of a PE programme in these patients are scarce [14–17], and have limitations in their applicability since they have not included a control arm or the PE has not been supervised [14, 15], nor performed using a progressive overload programme to optimise lean body mass gain [16]. The most recent Head and Neck Cancer Survivorship Consensus guidelines establish that further research is needed on the implementation of physical activity promotion in routine clinical care and the optimal timing, intensity and duration of exercise intervention in relation to oncological treatment, as well as research on the links between exercise and HNC recurrence or survival [18].

In this context, this study aims to evaluate the effectiveness of an innovative supervised PE programme to mitigate the loss of lean body mass, improve functional capacity and quality of life, which are dramatically reduced in people diagnosed with head and neck cancer; to compare it with the prescription of PE carried out independently; and to identify the optimal moment to apply this programme: before (prehabilitation) or after radiotherapy treatment.

## Methods/design

### Trial design

The SEHNeCa project is a multicentre, randomised, controlled clinical trial with three arms. Patients are recruited by the radiation oncologist at the patient’s first visit to the radiation oncology department, where they are asked to participate in the programme. Four assessments are made of all the participants, regardless of the group they belong to, at 0, 3, 6 and 12 months.

The baseline assessment will be performed during the same week as recruitment. After randomisation, the pre-radiotherapy group will start the supervised PE programme (at least 7–10 days before starting radiotherapy). The control and post-radiotherapy groups will initially receive physical activity guidelines that they can perform independently. The post-radiotherapy group will start supervised training 4–6 weeks after finishing radiotherapy and the control group will not have supervised training.

**Fig. 1:**
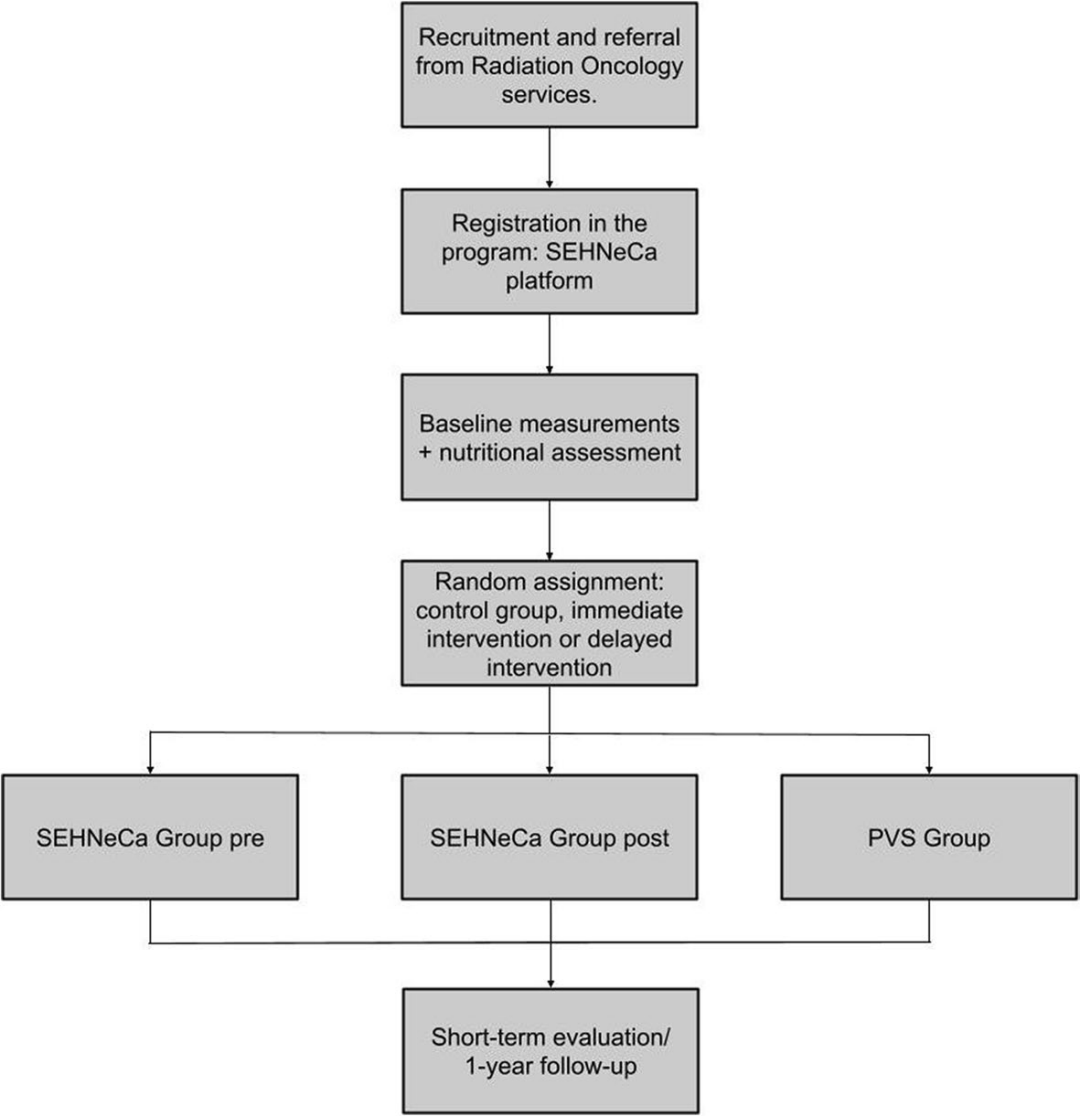
Flow diagram. *PVS* Prescribe Vida Saludable

All the patients will receive follow-up by the endocrinology service (before radiotherapy, during radiotherapy, at the end of radiotherapy and one month, 3 months, 6 months and 12 months after the end of radiotherapy) in which they will receive screening and assessment of nutritional status as well as individualised nutritional intervention following international guidelines [19], which may include nutritional advice, oral nutritional supplementation, and/or insertion of a feeding tube as required by each patient (Table 1) (Fig. 1).

**Table 1:** SEHNeCa Programme timeline

Follow up	D0	W1	W2		W3		W4		M2		M3		M4	M5	M6	M7	M12	M18
**Diagnosis and recruitment**	**X**																	
**Nutritional evaluation**		**X**									**X**				**X**		**X**	**X**
**Radiation oncology evaluation**	**X**										**X**		**X**		**X**		**X**	**X**
**Radiotherapy**					X													
**Measurements**		**X**											**X**			**X**	**X**	
**Supervised Exercise Intervention**			**PRE-RADIOTHERAPY GROUP**															
													**POST-RADIOTHERAPY GROUP**					
**Autonomous Exercise Intervention**			**Control Group and Post-radiotherapy Group**										**Pre-radiotherapy Group and Control Group**					

*D* Day, *W* Week, *M* Month

To achieve both these objectives, the radiotherapy-oncology and endocrinology departments of Basurto University Hospital, Cruces University Hospital and Galdakao Hospital have collaborated.

### Participants

*Inclusion and exclusion criteria* (Table 2).

**Table 2:** Inclusion/Exclusion criteria

Inclusion criteria	Exclusion criteria
Diagnosis of carcinoma of the larynx (except T1-T2 N0 of the glottis), pharynx, oral cavity, salivary gland, paranasal sinuses, or cervical metastasis of unknown origin in stages I-II-III-IVa-IVb	Decompensated heart disease, uncontrolled hypertension (SBP > 200 or DAT > 110), heart failure (NYHA > I), or constrictive pericarditis
Radiotherapy (with or without concomitant chemotherapy) preceded or not by surgical treatment and performed with radical intent	**Medical contraindication against exercise** (such as bone fractures, tendon ruptures, spondylolisthesis > 25% with neurological compromise, rhabdomyolysis, herniated disc in the spine with neurological symptoms, cardiovascular comorbidities such as unstable angina, current myocarditis or thrombophlebitis); Neutropenia (< 500 mm3), severe anaemia (Hb < 8.0 g/dl), platelet count < 50.000 microL
> 18 years	Radiotherapy with palliative intent
BMI > 18.5	Pregnancy
ECOG 0–1 or Karnofsky Index ≥ 80%	**Regular physical activity** (150 min/week of moderate activity or 75 min/week of vigorous activity, including two or more weekly strength training sessions), as measured by the PVS questionnaire
Signed consent form	Carcinoma of the glottis (T1-T2, N0)

*T*_*(1–2)*_ Tumour (TNM classification), *N*_*(0)*_ Node (TNM classification), *SBP* systolic blood pressure, *DAT *Diastolic blood pressure, *NYHA* New York Heart Association, *Hb* Haemoglobin, *BMI* Body Mass Index, *ECOG* Eastern Cooperative Oncology Group, *PVS* Prescribe Healthy Life

### Randomisation

Randomisation is carried out on a central basis through the coordinating committee. This will be made up of the organization responsible for the study’s management and the researchers. The committee will be blind to the assignment of patients in the comparison groups and will convene in the Primary Health Research Unit of Bizkaia.

The 120 patients included in the study will be randomly assigned and consequently registered and attached to one of the three study groups in a 1:1:1 ratio, through randomisation by random blocks of 3 or 6 patients. Patients are stratified according to HPV status and previous surgery yes/no.

### Evaluation

Evaluation of the SEHNeCa exercise programme involves measuring the safety, adherence, and effectiveness of the programme. These analyses incorporate elements of the reach, effectiveness, adoption, implementation, and maintenance (RE-AIM) framework [20].

### Safety

The incidence and severity of any adverse events (e.g. falls or muscle strains) that occur during the health-centre based sessions will be monitored and reported by the supervising exercise physiologist/nurse using programme-specific documentation.

### Adherence

Attendance at health-centre based exercise sessions and the reason for any missed sessions will be tracked throughout the programme. Further, completion of assessments at pre-programme and post-programme time points as well as follow-up questionnaires will be reported on.

### Effectiveness: primary study outcomes

Change in Lean Body Mass (kg), measured by using an Inbody 770 bioimpedance analyser (In-body, Seoul, Korea).

### Effectiveness: secondary study outcomes

#### Anthropometry and body composition

Height will be measured using a wall stadiometer, and body composition with a bioimpedance analyser. BMI (body mass index) (kg/m2), skeletal muscle mass (kg), fat free mass (kg); bone mineral content (kg), body fat mass (kg), body fat percentage (%), intra and extracellular water (l); extracellular water/total body water, visceral fat (cm2), basal metabolic rate (kcal) and phase angle (º) will be registered. Abdominal and waist circumference will be taken [21].

#### Physical function

Physical function is assessed by 400-m walk tests in a 20-m corridor in a controlled environment (temperature, 20-21º C; relative humidity, 50–55%; barometric pressure, 755–765 mmHg). The participants will be seated for measuring heart rate and blood pressure before and after the test. The repeat chair stand test (5 times sit to stand test), the handgrip dynamometry test and the Timed Up and Go (TUG) test will be carried out.

Maximal strength in the upper and lower body will be measured in terms of the 5-repetition maximum (5RM) test (the maximum load that can be lifted five times) in chest press, seated rowing and leg extension, respectively. These assessments are to be conducted by an independent exercise physiologist not involved with administering the exercise intervention.

#### Patient-reported outcomes

A series of questionnaires with sound psychometric properties are to be used to assess general health and cancer-specific quality of life at baseline and 3–6-12 months thereafter. The Medical Outcomes Study 36-Item Short-Form Health Survey (SF-36) will be used to assess general health-related quality of life status. Cancer-specific quality of life is evaluated by the core quality of life (QLQ-C-30) questionnaire developed by the European Organization for Research and Treatment of Cancer (EORTC). The Head and Neck cancer-specific questionnaire (H&N-35) is a supplementary module of the QLQ-C-30 questionnaire that includes 35 head and neck cancer-specific items. Finally, the Spanish version of the Shoulder and Pain Disability Index (SPADI) is a self-administered questionnaire that covers two aspects, one for pain and the other for functional activities, with lower scores representing less pain or disability.

### Supervised exercise intervention

The programme operates throughout the year and is a free 12-week, small group exercise programme supervised by specially trained instructors including exercise physiologists, physiotherapists and nurses. Participants are required to participate in supervised exercise sessions three times a week in the exercise laboratories, (primary health-care centres and Cruces University Hospital/Biocruces Bizkaia Health Research Institute). Twice a week, the sessions include a combination of strength exercises and moderate-to-high intensity aerobic exercise, and they last approximately 1–1¼ hours. The third session does not include aerobic exercise, and lasts 30 to 45 min. Strength exercises are conducted with free-weights (to provide a quantifiable measurement of progressive overload), elastic bands and suspension training, with the intention that the patients should learn how to train on their own. [22].

The strength exercises target five of the major upper and lower body muscle groups, as well as core exercises. Progression from static exercises towards more dynamic exercises is encouraged, aiming to activate more muscle mass. Among the main parameters that can be modified, the actual number of repetitions performed in a set, in relation to the maximum number that can be completed (i.e., proximity to muscle failure), recently called “level of effort”, will be used to individualise the strength exercise intensity, maximise the suitability of the exercise for each patient, and optimise induced neuromuscular fatigue [23, 24].The target intensity is adjusted from 9 to 12 repetitions out of the 18 repetitions that could be completed (written as 9–12 [18]), which is equivalent to ~ 60% of 1 repetition maximum (1RM) using 2 sets during the first 4 weeks, to 4 sets of 7–10 [14], which is ~ 70% of 1RM in the last 4 weeks of the programme.

**Fig. 2:**
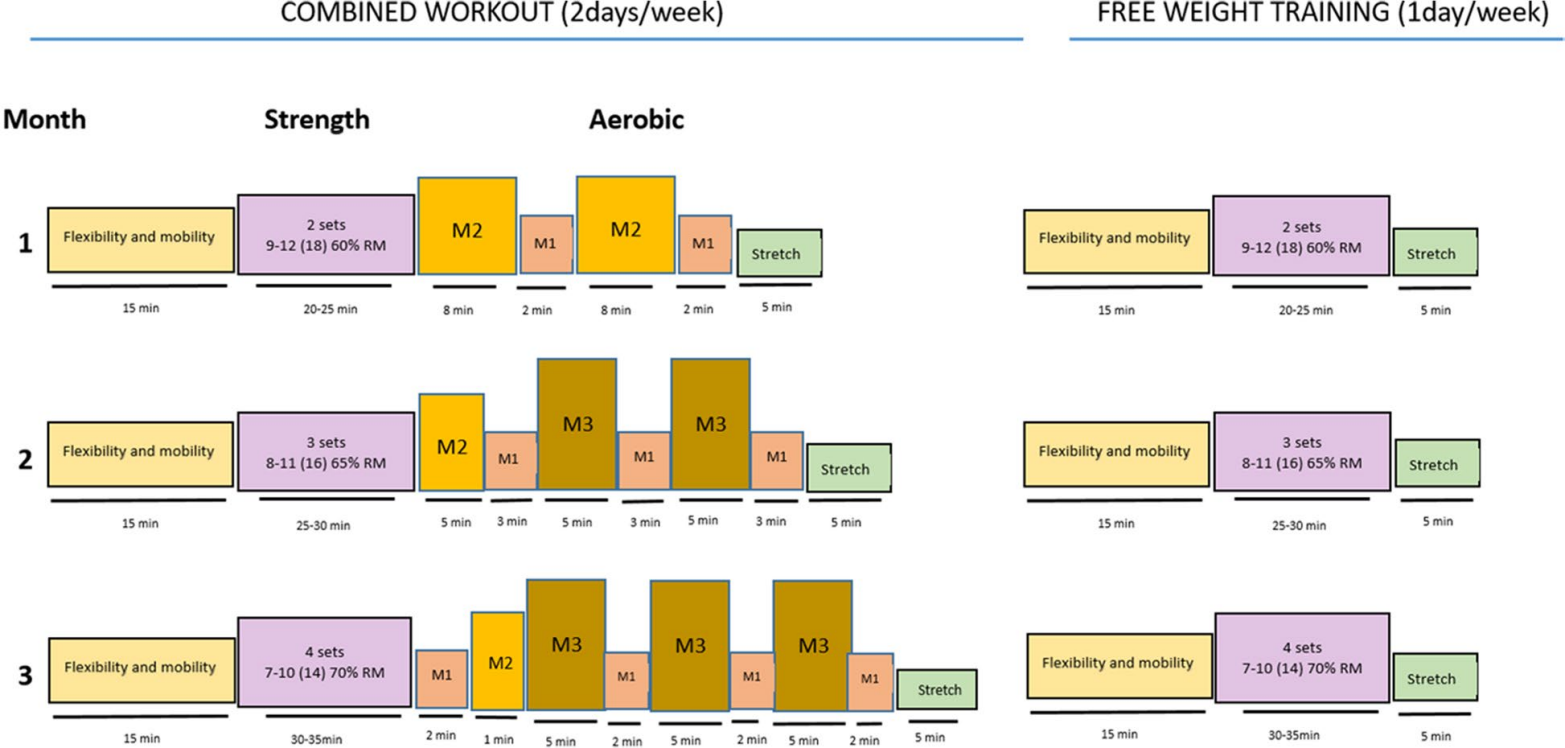
SEHNeCa exercise programme. M1: Low to moderate intensity; M2: moderate intensity; M3: intensive moderate intensity; 1RM: 1 maximum repetition

The aerobic exercise component includes 20–25 min of at least moderate intensity for 8-min periods alternating with 2-min lower-intensity periods during the first month, moving toward higher-intensity 5-min intervals by the third month (Fig. 2). The exercise intensity zones are tailored to each patient by the estimated maximum heart rate using the Eq. (220—age) [25] and applying specific intensity boundaries based on the heart rate reserve (HRR), defined as the difference between the resting heart rate and maximum heart rate. %HRR has been adopted by the American College of Sports Medicine as the gold standard for indirect assessment of exercise intensity [26, 27]. Every exercise session will be monitored with a heart rate monitor, teaching the patients to self-manage their exercise sessions with respect to the prescribed target intensities; the target intensity is between 40 and 85% of HRR [26, 27]. The perceived level of effort is to be recorded using the Borg Rating of Perceived Exertion (RPE) scale from 0 (rest) to 10 (maximal effort) [28], with the target intensity progressively increasing from 3 to 5–6 points (Table 3).

**Table 3:** Exercise intensity categories

Zone^a^	%HRR^b^	RPE^c^	Training type
High intensity training	> 85	> 5.5	High intensity training. The patient nears exhaustion and is no longer in a steady state
M3	60–84	4.5–5.5	Moderate to intensive training. The patient has difficulties in talking and sweating increases
M2	40–59	3–4	Moderate intensity. The patient notices an increased respiratory rate
M1	20–39	1–2.5	Low to moderate intensity. The patient does not notice any increase in the respiratory rate
Low-intensity training	< 20	< 1	Low intensity. This involves daily activities requiring low levels of effort

^a^The intensity zones are based on the estimated maximum heart rate and applying specific intensity boundaries based on HRR (43,44)

^b^HRR: heart rate reserve. Maximum heart rate—resting heart rate

^c^RPE: rating of perceived exertion. Determined using the Borg scale from 0 to 10 (45)

### Autonomous exercise intervention

Every patient in the control group will receive the corresponding standard treatment (i.e. radiotherapy), determined by the regular oncological consensus committee. In addition, they will receive a prescription concerning healthy lifestyles and a personalised exercise plan, together with an evaluation and individualised nutritional intervention by a physician at the Endocrinology and Nutrition department in Cruces, Basurto or Galdakao Hospitals, in the same way as for intervention groups. They will also be evaluated in the radiation oncology department with the routine follow-up (end of radiotherapy, one, three, six and twelve months after radiotherapy) to assess outcomes and toxicity due to oncological treatment according to Radiation Therapy Oncology Group and Common Terminology Criteria for Adverse Events (vs 5.0) toxicity criteria.

### Statistical analysis

Differences between treatment groups in changes in outcome variables will be estimated on an intention-to-treat basis. We will use linear mixed models for longitudinal analysis of repeated measurements of continuous outcomes (SAS PROC MIXED) and generalised logistic mixed models for dichotomous outcomes (SAS PROC GLIMMIX), considering intercept and time courses as random effects and testing the significance of the interaction of time slopes for each treatment group. No imputation method will be used to handle missing data, as longitudinal mixed-effect models based on maximum likelihood estimation are more appropriate for handling missing data than common imputation methods such as the last observation carried forward, complete case analysis, or other possible forms of imputation [29].

A sample size of 40 patients per group (120 in total) will give 80% power to the study to detect as significant (0.05) the difference at six months in a mean lean body mass loss of 25% between the control and rehabilitation groups, and of 12% between the rehabilitation and prehabilitation groups, corresponding to an estimated mean lean body mass loss from baseline of 4.8 kg (9.1%) in the control group, 3.6 kg (6.8%) in the rehabilitation group, and 3.2 kg (6%) in the prehabilitation group (standard deviation 2,4 kg). With an anticipated drop-out rate of 20%, we plan to enrol 144 patients (48 per group).

During these first 12 months of recruitment, the radiation oncology departments of Basurto and Cruces University Hospitals have attended 161 eligible patients, 112 met the inclusion criteria, of whom 63.4% agreed to participate in the study. Therefore, we expect that the recruitment period will last 24 months.

### Quality control

To ensure the quality of the study data, maximise the validity and reliability of the programme, and accurate measurement of the variables, we will take the following steps:

- Produce documents for the study, including operational manuals for fieldwork and forms for registering measurements and details of the intervention.
- Store all documentation (informed consent forms, documents containing results, etc.) in locked cabinets and on a database on a secure server.
- Hold regular meetings for quality control and write up progress reports every 3 months.
- The coordinator will contact the health centres daily, and report to the principal researcher every week.

## Discussion

The SEHNeCa programme aims to increase knowledge about the effectiveness and optimal start time of a supervised PE programme specifically targeted at patients with head and neck cancer, administered before, during or after radiotherapy, carried out at the primary care facilities and Cruces University Hospital.

The results obtained in the pilot study demonstrate the feasibility and safety of the project, showing that it is achievable in this type of patient. Additionally, through the pilot, good compliance with the circuit (radiation-oncologists, endocrinologists, evaluators) has been verified prior to the start of the training sessions, showing it to be feasible and achievable within the established deadlines. The pilot was only carried out with the group that performed PE prior to starting the radiotherapy treatment. This is due to the timing of the entire process, since patients must start the PE at least 7–10 days before the start of radiotherapy.

However, as the treatment progressed, there was a decrease in attendance at the PE sessions, mainly caused by the effects of the treatment. For this reason, it is necessary to verify the effectiveness with a larger sample, as well as the different effects of a PE programme before and during radiotherapy.

Moreover, both prehabilitation and rehabilitation programmes are limited by a lack of resources, awareness of the benefits of PE, and a lack of physical activity experts in cancer units [22]. For this reason, it is essential to have PE laboratories, with qualified professionals, in hospitals and outpatient centres, as proposed by the SEHNeCa project, so that this type of programme is more accessible to the entire targeted population and implantable in daily clinical practice.

### Strengths of the study

The main strength of the study is that it is a randomised controlled clinical trial, and therefore it includes a control group with which to compare the intervention groups. In this way it will be possible to determine the effects of the SEHNeCa programme more precisely. Furthermore, in the randomised clinical trials carried out to date, no group performs the PE intervention prior to the start of treatment. Therefore, the data resulting from the study can contribute to pinpointing the optimal moment for implementation of PE during the treatment process. Follow-up after treatment to assess acute/late toxicity will allow us to know the response to both the exercise programme and its adherence. 71 patients have been included in the study since the beginning of project recruitment in January 2022.

### Limitations of the study and contingency plans

One possible limitation could be the distance of the PE laboratory for some participants. Ideally, patients should be able to complete the supervised PE programme at their nearest health centre. Although we cannot definitely count on such resources, we might be able to offer availability in several centres so that they can use the laboratory of their choice.

One of the challenges we face is to improve patient participation and adherence to the programme. To do this, we will try to involve the patient in their recovery, and personalise the programme. Likewise, we consider it important to seek incentives, such as patients receiving feedback on their progress in the programme.

Finally, the circuit created to attract, evaluate and include the participants in the project is complex, since we have very little time to carry out the initial evaluations in radio-oncology, endocrinology and functional assessments, which could be another limitation. However, the supervision of the participants is continuous and comprehensive, also providing a large amount of data that can help the scientific dissemination of the project and for the design and implementation of new PE programmes.

### Ethical considerations

This study protocol complies with the Declaration of Helsinki and its amendments, as well as with good clinical practice. The Ethics Committee of the Basque Country approved the study in the health centres, ensuring it would be implemented in compliance with the established regulations. Regarding data confidentiality, only the study researchers have access to the data of the individuals who agree to participate in the study, in compliance with the Organic Act 03/2018 of December 2018, on the protection of personal data.

## Data Availability

The datasets used and/or analysed during the current study are available from the corresponding author on reasonable request.

## Abbreviations

HNC: Head and Neck Cancer
HPV: Human Papilloma Virus
HRR: Heart Rate Reserve
PE: Physical Exercise
RM: Repetition Maximum
SEHNeCa: Supervised Exercise for Head and Neck Cancer
